# The Association between Alcohol Dependence and Depression before and after Treatment for Alcohol Dependence

**DOI:** 10.5402/2012/482802

**Published:** 2012-01-26

**Authors:** Mary W. Kuria, David M. Ndetei, Isodore S. Obot, Lincoln I. Khasakhala, Betty M. Bagaka, Margaret N. Mbugua, Judy Kamau

**Affiliations:** ^1^Department of Psychiatry, University of Nairobi, P.O. Box 74846-00200, Nairobi, Kenya; ^2^Department of Psychiatry, University of Nairobi, P.O. Box 48423-00100, Nairobi, Kenya; ^3^Department of Psychology, University of Uyo, P.O. Box 4230, Uyo, Nigeria; ^4^Department of Psychiatry, University of Nairobi, P.O. Box 59176-00200, Nairobi, Kenya; ^5^Crisis Pregnancy Ministry, Nairobi, Kenya; ^6^Harmony Psychological & Mental Health Consultancy, Nairobi, Kenya; ^7^Kitale District Hospital, Ministry of Health, P.O. Box 4463-300, Kitale, Kenya

## Abstract

The presence of depression in alcohol-dependent persons is likely to influence treatment process and outcomes. Identification of depression is important though not every depressed alcohol-dependent person requires treatment with antidepressants. Understanding the association between depression and alcohol dependence is essential for proper management of alcohol dependence. *Objectives*. To determine the prevalence of depression among alcohol-dependent persons before and after alcohol detoxification and rehabilitation. *Design*. Clinical trial with pre-/postmeasurements. *Method*. The CIDI and WHO-ASSIST were administered to 188 alcohol-dependent persons at intake and after six months. A researcher-designed sociodemographic questionnaire was also administered at intake. *Results*. The prevalence of depression among alcohol-dependent persons is high (63.8%) with a significant association between depression and the mean AUDIT score. At posttest, depressed participants had a statistically significant craving for alcohol. *Conclusion*. Alcohol dependence is associated with major depression.

## 1. Introduction

The prevalence of comorbidity of depression and alcohol use disorders (AUD) has been demonstrated in a number of researches [1–4]. Depression in an alcohol-dependent person has been reported to not only lower the resolve to resisting alcohol use, but may also lead to use of alcohol to relive the depressive symptoms [5, 6]. It is important to understand the significance of cooccurrence of depression and alcohol use disorders since this may explain why majority of cases relapse after treatment for alcohol dependence [5, 6]. In addition it may explain why antidepressants have been shown to moderately benefit patients with both depression and alcohol use disorders [7].

In Kenya a study by Ndetei et al. showed that there was positive correlation between major depressive illness, panic disorder, and alcohol abuse among patients admitted at the main referral psychiatric hospital [8]. Although the cooccurrence of depression and alcohol use disorders has been confirmed by several studies, the relationship between the two disorders has been difficult to describe [9]. This cooccurrence is at prevalence rate of 16%–68% [10]. Studies have attempted to differentiate between depressed and nondepressed alcohol-dependent persons with particular focus on the participant's level of alcohol dependence, demographic characteristics, or illness-related variables. It has been shown that depression is more related to the current alcohol drinking episode than lifetime diagnosis of depression [11]. Depression diagnosed in the current episode of alcohol dependence normally remits after 2 weeks of detoxification and abstinence and falls to normal range within 3 weeks [11, 12]. The rapid recovery is in contrast to the slower (17 weeks) recovery from a major depression [13]. This may imply that depression in alcohol dependence is as a result of effects of chronic alcohol intoxication and is related to the recent episode of drinking.

There are two possible explanations for the association between alcohol use disorders and major depression; firstly it may be that both disorders have common underlying genetic and environmental factors that jointly increase the risk of both disorders. Secondly, the two disorders may have a causal effect with each disorder increasing the risk of developing the other.

A number of studies have found evidence of a persistent association between alcohol use disorders and major depression, even after controlling for confounding factors [14, 15].

Flensborg-Madsen et al. in a prospective study showed that the causal role of alcohol use disorders in major depression was stronger than the causal role of major depression in alcohol use disorder [16].

Literature suggests that the more a person drinks the more they are likely to develop major depression [17]. Presence of alcohol use disorder or major depression is associated with a double risk of either disorder. There is a moderate association between the two [18]. Causal relationship between Alcohol use disorder and depression has been attributed to AUD causing depression or depression causing AUD [19]; in the latter the person uses alcohol to relieve the depressive symptoms. In addition there may be a reciprocal causal relationship where the presence of alcohol use raises the possibility of developing the other disorder [2, 5, 14, 20–25]. Individuals, who drink alcohol to reduce emotional stress, may be self-medicating themselves with alcohol [26–28], and a link has been shown where depression predicted alcohol use disorder and alcohol use disorder predicted depression [29].

The causal effect of AUD leading to depression implies that some cases of depression resolve after treatment of alcohol dependence [12, 30–33]. Persons that use alcohol to relieve depressive symptoms may require treatment for depression to achieve full remission after alcohol use disorder treatment [18].

Various studies have shown both a metabolic and neurophysiological link between alcohol use and depression [34–42]. While some studies have reported gender differences in the link between alcohol use disorders and depression [43–46], others have not [47].

There is no published data from Kenya on the association between alcohol use disorders and depression. Research-based evidence would prompt clinicians to screen for depression in alcohol-dependent persons. 

## 2. Method 

The objective of the study was to determine comorbid depression among 188 Alcohol Use Disorder Identification Test (AUDIT) [48] positive participants. The data is part of that collected in a six-month prospective study done to determine the cost-effectiveness of community-based and institution-based detoxification and rehabilitation of alcohol-dependent persons. The sample was purposefully selected. The study was conducted at the Kangemi informal settlement located in the west of Nairobi city in Kenya in 2008. The study area has a high population of people with use disorders, particularly alcohol dependence [37]. 

Consenting participants aged 18 years and over were included in the study if they were alcohol-dependent with an AUDIT score of 15–40 (for males) and 13–40 for females. Persons aged 18 years and over are legally eligible to consent. Consent explanation was given before the participant gave consent. All aspects of detoxification and rehabilitation including medication, dosage and side effects, and right to withdraw at any time during study were explained after which consenting individuals signed a consent. Ethical approval was obtained from the Kenyatta hospital/University of Nairobi research and ethical review board. Excluded from the study were those unavailable or unwilling to join the study for the 6 months. Those suffering from severe medical and neuropsychiatric complications (including delirium tremens, active psychosis (hallucinations, delusions), suicidal thoughts and tendencies, and severe memory difficulties) at time of screening for intake were also excluded. Those with multiple drug use/abuse were included in the study. A total of 20 persons were excluded.

A researcher-designed sociodemographic questionnaire (SDQ) was administered at intake to provide necessary information including that which was needed for followup of participants. Alcohol Smoking Substance Use Identification Screening Test (ASSIST) [49] was used to screen for alcohol and other substance use and alcohol-related problems. The PAPI version of the Composite International Diagnostic Interview (CIDI) [50] instrument was administered to screen for psychiatric comorbidity. Both the ASSIST and the CIDI were administered both at intake and at six months. 

The participants were subjected to alcohol detoxification for 10 days using a pair of ampoules of Pabrinex 1 & 11 given by intravenous injection daily for 3 consecutive days, Diazepam 5 mg and Carbamazepine 200 mg for 5 and 10 consecutive nights, respectively, on outpatient basis at intake. Pabrinex is parenteral high-potency Vitamin B and C combination. Although the study participant had a general physical examination done (including blood pressure, temperature, and body weight check), no laboratory or radiological investigations were done in the current study. Low doses of benzodiazepine were given to all participants to avoid heavy sedation that would complicate existing medical conditions. Similarly low doses of Carbamazepine were used.

There was documented followup at home for each participant by the community-based health worker (CBHW) twice a week, and the principal researcher (P.I) or assistant (once a week) at Kangemi Heath Centre for a period of 6 months. A follow-up questionnaire was used to determine whether individual was abstaining from alcohol and symptoms that they were experiencing. This was filled once a week by the principal researcher and twice a week by the CBHW. Both the P.I and the CBHW reports on the drinking status of the participants were compiled weekly. Any discrepancy between the two structured reports was confirmed by a home visit by the CBHW.

There was a bimonthly group therapy conducted in groups of 20 s by the P.I and a clinical psychologist.

## 3. Data Analysis 

Data collected was coded, entered, and stored in computer. Only the PI had the name related to the code number. The data was analyzed using STATA version 10 and descriptive and inferential statistics performed. 

A logistic regression analysis was done to determine factors associated with presence of depression. The data obtained by use of the composite international diagnostic interview was analyzed for major depression.

## 4. Results

A total of 188 participants underwent community-based detoxification but only 156 were followed up for the six months. Majority (91.5%) were male and 8.5% were female. Majority (60.5%) of the participants had begun drinking alcohol before the age of 18years, with the mean AUDIT score being 28.6 for male and 26.6 for females. The mean age of the group was 31.9 years, with majority (84%) of the participants aged below 40 years. The majority (53.3%) of the participants earned an income of less than 143 United States dollars per month. The majority (51.1%) were married, while 38.9% were single. The remaining participants were either separated or divorced. 

The prevalence of depression at intake before detoxification was 63.8% (120 participants). Six months after detoxification and completion of rehabilitation the prevalence of depression was 30.2% (47 participants).There was a statistically significant reduction (*P* value 0.000) in the prevalence of depression at six months during which period the participants had undergone community-based detoxification and rehabilitation for alcohol dependence. Three participants were referred for treatment of major depression within the period of the study. 

There was a statistically significant association (*P* value 0.002) between depression and the level of alcohol dependence at intake. Participants with an AUDIT score of 19 and above were more likely to be depressed. There was no statistically significant association between depression and sociodemographic characteristics. 

Analysis of data collected at the end of the sixth month showed a statistically significant association between depression and alcohol use (*P* value 0.02). In addition those who were depressed at six months had more severe craving for alcohol than those who were not depressed (*P* value 0.03). 

Polysubstance use was noted, with 50% of the participants using tobacco while 21.3% of them were using cannabis. Less than 1% of the participants used other substances of abuse.

## 5. Discussion

Several studies have demonstrated the extent of comorbidity between depression and alcohol use disorders [1–4]. The current study confirms the high prevalence rates (63.8%) of major depression among the alcohol-dependent persons. This is close to a higher limit (68%) of the estimated prevalence of cooccurrence of depression and alcohol dependence [10]. Although only 3 participants were referred for treatment of depression there was a statistically significant reduction of the comorbidity after treatment of alcohol dependence in the current study. The National Institute on Drug Abuse (NIDA) has concurrent treatment for comorbid disorders as one of its fundamental principles of substance-induced disorders [51].

The current study did not investigate the cause of association between depression and alcohol dependence. The finding of a high prevalence rate of depression among the study participants calls for the need to evaluate persons for depression before and after alcohol dependence treatment. In addition it is important to obtain family history of mood disorder and life time diagnosis of depression since the presence of such histories puts the individual at a greater risk of developing major depression. 

Some of the study participants were still depressed six months after alcohol detoxification. There was a statistically significant association between being depressed at six months and alcohol use at posttest. Possibly some of the depressed persons were using alcohol to relieve their depressive symptoms, and major depression was their, primary diagnosis. Literature indicates that persons that use alcohol to relieve depressive symptoms may require treatment of depression to achieve full remission after alcohol use disorder treatment [18]. Secondly, it is possible that continued use of alcohol by these participants may have sustained the depression. Research indicates that the more a person drinks the more they are likely to develop major depression [17], and presence of either AUD or major depression is associated with a double risk [18]. This raises the question as to whether all comorbid depression in alcohol-dependent persons should be managed with antidepressants and explains why antidepressants have been reported to only exert a modest beneficial effect for patients with combined depressive disorder and alcohol dependence [7].

The need for screening for depression in alcohol-dependent persons and continuous monitoring for it during treatment of alcohol dependence cannot be overemphasized. This is because untreated persistent depression may reduce the resolve to refrain from alcohol, or alternatively depression may lead to self-medication with alcohol [5, 6]. This may explain why relapse rates are high after treatment for alcohol dependence. 

Other factors, that may contribute to high relapse rates after treatment for alcohol use: disorders, includes multiple-drug use: in particular the use of cannabis is likely to further complicate the treatment of alcohol dependence due to lack of motivation.

The current study, like another study [2], did not show gender link between alcohol use disorders and depression. There was however a statistically significant association between being depressed and craving for alcohol at six months. Craving for alcohol is associated with a desire to use the alcohol, and those with severe craving are more unlikely to stop drinking alcohol as compared to those with mild craving. This association between depression and craving may necessitate those individuals who are still depressed after alcohol detoxification and rehabilitation receive antidepressants to possibly reduce the chances of relapse to alcohol use.

## 6. Limitation

The study sample was one of convenience, purposely selected for alcohol detoxification and rehabilitation. Secondly no past psychiatric history of depression or family history of mood disorders was obtained from the participants at intake. Such a sample may produce skewed prevalence rates of depression.

## 7. Conclusion

1. The prevalence of depression among alcohol-dependent persons is high.
2. There is recovery from depression after alcohol detoxification and rehabilitation, and majority of the cases do not necessarily require treatment for the depression.
3. In addition persons that are depressed have a significantly higher craving for alcohol after detoxification and rehabilitation.
4. It is important to screen for depression and evaluate to determine the treatment needs during detoxification and rehabilitation.
